# Bundling occupational safety with harm reduction information as a feasible method for improving police receptiveness to syringe access programs: evidence from three U.S. cities

**DOI:** 10.1186/1477-7517-6-16

**Published:** 2009-07-14

**Authors:** Corey S Davis, Leo Beletsky

**Affiliations:** 1 University of North Carolina, Gillings School of Global Public Health, Campus Box 7411, Chapel Hill, North Carolina 27599, USA; 2 Yale University, Center for Interdisciplinary Research on AIDS, 135 College Street Suite 200, New Haven, CT 06510, USA

## Abstract

**Introduction:**

In light of overwhelming evidence that access to sterile injection equipment reduces incidence of injection-attributable bloodborne disease without encouraging drug use, many localities have authorized sterile syringe access programs (SAPs), including syringe exchange and pharmacy-based initiatives. Even where such interventions are clearly legal, many law enforcement officers are unaware of the public health benefits and legal status of these programs and may continue to treat the possession of injection equipment as illegal and program participation as a marker of illegal behavior. Law enforcement practice can impede SAP utilization and may increase the risk of needlestick injury (NSI) among law enforcement personnel. Many SAPs conduct little or no outreach to law enforcement, in part because they perceive law enforcement actors as unreceptive to health-promotion programs targeting drug users.

**Case description:**

We report on a brief training intervention for law enforcement personnel designed to increase officer knowledge of and positive attitudes towards SAPs by bundling content that addresses officer concerns about infectious disease and occupational safety with information about the legality and public health benefits of these programs. Pilot trainings using this bundled curriculum were conducted with approximately 600 officers in three US cities.

**Discussion and evaluation:**

Law enforcement officers were generally receptive to receiving information about SAPs through the bundled curriculum. The trainings led to better communication and collaboration between SAP and law enforcement personnel, providing a valuable platform for better harmonization of law enforcement and public health activities targeting injection drug users.

**Conclusion:**

The experience in these three cities suggests that a harm reduction training curriculum that bundles strategies for increasing officer occupational safety with information about the legality and public health benefits of SAPs can be well received by law enforcement personnel and can lead to better communication and collaboration between law enforcement and harm reduction actors. Further study is indicated to assess whether such a bundled curriculum is effective in changing officer attitudes and beliefs and reducing health risks to officers and injection drug users, as well as broader benefits to the community at large.

## Introduction

The spread of bloodborne disease through injection drug use is a longstanding problem in the United States and abroad, with syringe sharing as the primary modality for disease transmission among injection drug users (IDUs) [1,2]. Many states and localities have implemented syringe access programs (SAPs) to reduce the sharing of syringes, including over the counter syringe sales and syringe exchange programs (SEPs) [3]. These interventions have been associated with decreased incidence of bloodborne disease and risky syringe-related behaviors among IDUs. They have also been shown to increase access to drug treatment and to reduce the number of improperly discarded syringes in the community [4-7].

Injection-related disease transmission is largely an unintended consequence of laws aimed at curbing illicit injection drug use. Possession of injection equipment for the purpose of injecting illicit drugs is unlawful in many U.S. jurisdictions, severely restricting syringe access [8-10]. A recent study of 89 U.S. cities found that higher rates of three measures of legal repressiveness (hard drug arrests, police employees per capita and corrections expenditures per capita) were associated with higher HIV prevalence among IDUs [11]. Studies have repeatedly shown that laws restricting syringe possession raise street prices for injection equipment, lead to risky injection-related practices, and may contribute to improperly discarded syringes [9,12-14]. These laws also may increase the risk of officer needle stick injury (NSI) [8].

Even where the law has been amended to make SAPs clearly legal, officers may not be aware of these changes, may not understand the public health rationale for them, or may simply choose to ignore them because they perceive them as misguided or counterproductive [10,15,16]. As a result, officers may continue to arrest IDUs for possession of legal injection equipment, confiscate or destroy this equipment, or treat participation in SAPs as a marker of illegal behavior, thereby limiting the public health benefits of SAPs [11,16-22]. Such practices may also discourage IDUs from informing officers that they are in possession of injection equipment, thereby increasing the risk of officer NSI.

Although the precise extent of this problem is unknown, it is clear that law enforcement interference with SAPs is widespread. In a 2007 survey of syringe exchange programs in the United States, 29% of respondent programs reported police harassment of participants at or near their access point, while 8% reported police arrest or harassment of program staff [23]. Recent data from an ongoing national survey of SAP respondents in the U.S. suggest that syringe exchange programs experience an annual average of 11 incidents involving police harassment of clients and at least one incident of staff harassment. Four out of 85 programs sampled to date report police arrest of staff members over the previous 12 months, and almost one in five report at least monthly uninvited police visits to syringe exchange venues. About one third report police confiscation or destruction of clients' legal injection equipment over the same period (unpublished data on file with authors).

Many law enforcement officers – police, deputy sheriffs, probation/parole officers and other sworn personnel – routinely come into contact with people who use drugs, those with mental illness and other groups perceived as likely to commit crimes and disturb public order. These encounters can lead to searches, inventory of personal effects upon arrest and other activities that may increase officer occupational safety risk through NSI and other communicable pathways [24,25]. Although officers express significant anxiety about these risks, they typically have limited access to training and resources that can equip them to effectively reduce the risks and to navigate the complex set of mental health, addiction and other issues pervasive among IDUs and other marginalized populations. This can be a source of apprehension and miscommunication between these populations and officers, as well as a point of conflict between law enforcement and public health professionals [16,26-28].

### Disparate Cultures, Common Goals

Public health and law enforcement professionals tend to embody distinctly divergent views of drugs and drug users. Although there is a great deal of variation between and within members of each group, public health professionals are more likely to view drug addiction through a medical and social support lens that favors treatment and iterative approaches to illicit drug use [29]. Conversely, law enforcement officers tend to regard illicit drug use primarily as a legal and moral issue best resolved through rule-based, disciplinarian approaches rooted in deterrence, incapacitation, and punishment [10].

Both groups, however, have much in common. Both sectors take as their mandate the promotion and maintenance of healthy and safe communities. Both are predominantly comprised of people who chose the profession out of a sense of duty or desire to contribute to the health and welfare of society. From an institutional standpoint, both must compete for scarce public resources and are subject to often frustrating and competing directives from political leaders who may not understand or properly credit the complex realities faced by the organizations.

Despite the fact that public health and law enforcement professionals often work with the same populations in the same neighborhoods, there is relatively little formal institutional overlap between the two sectors [10,30]. In many communities, officers form the first and sometimes only point of contact for chronic IDUs and other groups engaged in criminalized behaviors. Because of this reality, harmonizing policing practice with evidence-based public health programs such as SAPs is an essential element of successful public health efforts directed at these groups.

In recognition of the potential for synergy between the two sectors, public health practitioners are increasingly forging partnerships with law enforcement that are mutually beneficial to those organizations as well as society as a whole [31-35]. Perhaps because they largely believe that such activities would be futile or a poor use of scarce resources, it appears that many SAP operators and other public health actors have failed to identify and adequately address the concerns of law enforcement in planning and implementing SAPs, and rarely emphasize the positive health benefits such programs may have for officers.

### Collaboration and Training to Change Police Response to Marginalized Populations

Even when they interpret a person's illegal behavior as stemming from a mental illness, officers may arrest that person if they perceive that no appropriate alternative to incarceration is available. This view may often be correct; in many jurisdictions, psychiatric treatment is more accessible in correctional settings than in the community [36,37]. Similarly, police crackdowns aimed at people who use drugs are sometimes justified by public officials as a means of enrolling drug users into treatment [38]. Qualitative research suggests that officers often view arrest and incarceration as fundamentally flawed approaches to reducing chronic injection drug use, but consider these tools to be the only ones readily available to them in addressing drug-related crime and nuisance [16].

Many similarities exist between officer reaction to individuals with mental illness and individuals who use illegal drugs. Both groups are stigmatized by the larger community and are often seen by police as likely to commit crimes, and police are more likely to use force with both groups than with most other populations [39]. Research has shown that law enforcement officers are interested in receiving training in improving their interactions with people with mental illness. A number of collaborative efforts between mental health advocates and law enforcement have been implemented, with positive effects on officers' attitudes, beliefs, and knowledge [32,37,40,41].

Collaborations between harm reduction and policing professionals, including officer training, have also shown promise. Internationally, Drug Action Teams (DATs) comprised of police, social services, and health providers that work to reduce drug-related crime while increasing access to effective drug treatment have been implemented in Great Britain and Australia to the benefit of police and IDUs [42,43]. In China, international funding has been utilized to create teaching materials, conduct attitudinal research, and train professors at Yunnan Provincial Police Academy in public health and the role of police in implementing harm reduction programs. As a result of these efforts, over 6,000 cadets and many senior officers have received harm reduction training including the role of police in facilitating harm reduction programs, and regular meetings are held between police and harm reduction actors [44]. Harm reduction training for police has also been conducted in other parts of East and Southeast Asia as well as the Ukraine [45,46].

Trainings directed at harmonizing law enforcement with public health activities have also been successfully implemented in the United States. In 2003, in response to an overdose epidemic among IDUs in San Francisco, a local harm reduction program initiated an intervention to distribute naloxone, a drug used to reverse opioid overdose, and train police and community members on its proper use. The program trained approximately 200 officers of all ranks about the goals and evidence base for the program and the chilling effect of the threat of arrest at overdose sites on the willingness of witnesses to intervene and summon emergency responders. After the training, no program participants reported being arrested for possession of naloxone or presence at overdose sites, although some participants reported having their naloxone confiscated by police [47]. Harm reduction and public health agencies in New York and New Mexico sponsor programs to train officers on SAP law and policy. These activities are not the norm, however; nationally, only about 20% of U.S. SEPs participate in police trainings (unpublished data on file with authors).

## Case description

While some harm reduction agencies have conducted either one-time or ongoing trainings for police on the benefits of SAP, many programs are reluctant or unequipped to conduct such outreach. We could find no reports in the peer-reviewed literature as to what methods are employed, and what results are achieved, by such trainings. Based on evidence from the fields of police training and adult learning together with our interactions with law enforcement actors in a number of jurisdictions, we came to believe that officers might be receptive to information regarding SAPs when it is bundled with information about reducing officer occupational safety and is delivered by a trusted source [48,49]. Our goal was to determine whether departments and officers would be receptive to such a bundled training in three diverse cities in the Eastern United States.

We identified and approached three departments (Pawtucket RI, Philadelphia PA and Wilmington DE) to conduct these feasibility trainings. As a point of departure for the design of the curriculum, modules used in training police in New York and New Mexico were utilized together with knowledge gained from informal discussions with officers and public health professionals. Curricula (available at ) were designed to be brief and easily adaptable. They covered the evidence base for SAPs, the legal status of these programs in the jurisdiction, and the benefits of SAPs to the occupational safety of law enforcement personnel as well as the wider community. The curricula were tailored for the geographic, policy, and other contexts of each city.

The trainings were delivered sequentially beginning in summer 2006. While each training was adapted to the local situation, they adhered to the same general model: the training sessions lasted less than 30 minutes, emphasized occupational safety, were delivered to officers at either the police station or police academy, and included members of the local SAP, who were on-hand to answer questions, further discuss the program and reiterate the benefits of the two groups working together to reduce drug-related harm.

### Site 1: Pawtucket, Rhode Island

In 2000, the Rhode Island Legislature decriminalized over-the-counter sales of hypodermic syringes. A qualitative study of police officers in the Pawtucket Police Department – a semi-urban setting – three years after passage of the law found that respondents were misinformed about the change, with a substantial proportion of officers self-reporting confiscating and destroying legal injection equipment in spite of the new law [16]. Interviews suggested that law enforcement personnel knew little of the syringe deregulation's public health underpinnings, which led to misinterpretation and a sense of hostility towards the law [16]. These data also highlighted the sense of anxiety and lack of training among the officers regarding infectious disease risk flowing from frequent contact with IDUs and occupational NSI events.

Using the demonstrated need from that study and the personal relationships that had been developed in the course of conducting the research, one of the authors (LB) approached the Pawtucket Police Department to suggest training officers about occupational safety, the law and SAPs. The findings were also presented to Rhode Island's Attorney General, who agreed to the need for training to address the disconnect between the law and police knowledge, attitudes and practices. With support from the department, LB applied for and received grant funding from the Rhode Island Foundation to conduct a pilot training for department personnel. LB then collaborated with the departmental training staff to design a training module to be delivered to the department's officers. The training module included a PowerPoint presentation, training evaluation forms, and presenter's notes.

Although funding was approved and the department agreed to participate in the training, the project was stalled for six months because the department's leadership had not filed a letter of agreement to serve as the site for the project, a key grant requirement. Once this requirement was cleared, LB met with personnel from the department's Planning and Training Division to finalize and pilot the curriculum. Based in part on the collaborative relationships that had been formed during the research phase, the Director of the Planning and Training Division agreed to engage his Division's staff in the implementation of the training program.

The training was presented by the staff of the Planning and Training Division to about 140 street-level officers in the department through 6 sessions between March 2006 and February 2007. Each session lasted 30 minutes and was delivered as part of an hour-long session that also included training on another, unrelated topic to lower personnel costs to the department. All participants were later provided with tactical needle-resistant gloves at no charge to the department or officers.

### Site 2: Philadelphia, Pennsylvania

SAPs were authorized in Philadelphia in 1992, and a legal syringe exchange program has operated for many years under contract with the city health department. However, a 2005 study found that use of the exchange fell significantly after the implementation of an anti-drug initiative launched by the city's police department in 2002 [50]. Although police management had issued an internal memorandum instructing officers not to target syringe exchange attendees, clients regularly reported that police officers impeded their efforts to reduce their bloodborne disease risk, and exchange staff (including author CD) had observed officers improperly discarding used syringes taken from exchange clients.

One of the authors (CD) together with other members of the SAP and county health department officials arranged meetings with supervisory police personnel in the area in which the SAP is located to discuss the ongoing negative impact of police action on access to the SAP and the connection between SAP access and reduced officer NSI risk. The feasibility of implementing a training similar to that conducted in Rhode Island was raised. The level of interest in participating in the initiative varied. One upper-level police official was unsupportive of the SAP, while another expressed wariness of possible negative repercussions he believed might occur if he was seen as collaborating with an organization that, while legal and receiving explicit governmental support, is still considered controversial and misguided among some officers and community members.

The Captain in command of the area in which the SAP office and largest outreach site are located, however, was supportive of the program and receptive to the idea of the bundled training. He suggested that CD contact the department's Infection Control Officer. The Infection Control Officer, a registered nurse and Sergeant in the department, was also receptive. It became clear through these interactions that many department personnel, both officers and command staff, were unaware that the SAP was legal, did not understand its role in community protection, and had not considered that it could reduce their risk of occupational NSI. We also became aware that officers received little training in reducing the risk of NSI, and were not provided with appropriate syringe disposal containers or barrier methods with which to handle and dispose of confiscated syringes.

Based on this information and with the support of the Captain and Sergeant, the SAP applied for and received grant funding from the Drug Policy Alliance to train officers in the police district in which the SAP is located, and to record the training for further dissemination. Trainings were conducted for all three shifts in the district over a single day in summer 2006 by a high ranking officer from the Albuquerque, New Mexico police department who had conducted similar trainings in New Mexico.

The training module was similar to that used in Rhode Island. Using a PowerPoint presentation, the New Mexico officer discussed the legality of the SAP and its ability to direct clients to treatment and other services and the direct benefits of the program to law enforcement. He also shared his personal experience of the positive impact SAP had on law enforcement in his department. As part of the training, the Infection Control Officer presented information on proper procedures for reporting and avoiding occupational NSI. Officers were provided with puncture-resistant gloves and approved syringe disposal containers to reduce their NSI risk. The SAP also provided the district office with a large syringe disposal container which is collected and replaced by the SAP at its expense. Approximately 90 officers and supervisory staff received the training.

### Site 3: Wilmington, Delaware

In June 2006, Delaware state law was modified to explicitly permit SEPs. This change provided an opportunity to build on the previous two training initiatives by designing and implementing an integrated curriculum to be presented to police concurrently with the change in syringe access law. The program was designed in collaboration with local and state public health and law enforcement officials. The trainings were coupled with programs for prevention, monitoring and response to law enforcement-related problems, and were augmented by trainings for SAP clients on interaction with the police. Funding for curriculum development and program activities was provided by the Drug Policy Alliance.

A Wilmington non-profit contracted with the Division of Public Health to operate the first syringe exchange program authorized under the new law. Before the program opened, staff members from this non-profit and representatives of the state public health agency met with the Wilmington police chief to discuss the effect of the change in law on police practice, and to suggest that training modeled on that conducted in the previous two cities might be beneficial. The chief agreed to the Department's participation and assigned the Officer in Charge to hash out a number of practical questions relating to the change in law and its effect on departmental action in relation to the SEP.

The training portion of the initiative, which is ongoing, was developed in collaboration with public health and law enforcement officials. The sessions are administered by SAP personnel, often accompanied by a training officer. The trainings include information on the basic design of the SAP, its geographic scope, the authorizing legislation, and the legal immunities the law affords to clients. The curriculum stresses the public health goals of the program and the specific ways they shape the department's standard operating procedures, including changes in search, arrest, and referral activities. It also describes proper occupational safety procedures for handling syringes, communication techniques for prevention of NSI, and appropriate actions to be taken in the case of an NSI. To date, the training has been delivered to approximately 175 city police, 100 county police and 100 state police officers. The training has also become a part of the standard curriculum at the county and city police academies, with 2 county and 1 city police academy cohorts receiving the training to date.

The training was coupled with education for exchange clients on their rights under the law. This system was implemented both at the exchange and at the local public defender's office, which pledged to add exchange-specific questions to its standard client intake form. Officers are also provided with a wallet-sized information card, which includes guidelines on avoiding NSI, information about the authorizing law, and a phone number officers can call with questions or referrals to the SEP.

## Discussion and evaluation

Simply changing the law to improve syringe access and reduce HIV risk may be insufficient to change key factors that shape such access, including police practice. Organizational inertia, lack of information and real or perceived lack of options can slow the dissemination, assimilation and implementation of new laws, policies and values [51]. Officers may not know about the policy change, or may simply ignore it as misguided [16]. Without alternative sources of information, officers rely on their superiors and peers – the workplace's implicit and explicit organizational values – for informational and attitudinal cues about drug use and drug users [51-53].

Without additional training, therefore, officers will tend towards the status quo. For training to be effective, it must be sufficiently tailored to change attitudes in the face of both inertia and oppositional organizational culture [51]. Trainings must clearly demonstrate to the officer why he or she should abandon the status quo in favor of novel procedures and practices. In the context of legal reforms to facilitate syringe access, this amounts to a new method of policing IDUs, one that may go against not only officer's existing beliefs but also organizational incentives (such as making arrests and appearing "tough on crime"). Although there is agreement that training is a vital element in changing officer behavior and attitudes, there is a lack of consensus as to the best method for achieving this change [54,28]. Recent scholarship, however, has shown that police culture is "much more open to change than was once assumed [55]."

In the realm of bloodborne disease prevention among IDUs, policing represents a key structural barrier to service uptake. In spite of its prominence, most harm reduction organizations do not systematically address interactions with law enforcement. Based on previous models, we set out to ascertain whether trainings that bundle the public health rationale behind SAPs with an explicit focus on officer occupational safety were feasible and would encourage receptivity among both street-level and supervisory law enforcement personnel. We collaborated with local public health and law enforcement officials to design and deliver such training in three American cities.

At all three locations, the trainings went forward with the approval and cooperation of law enforcement management. Although not all members of the departments were supportive of the SAPs, none of the three departments that were approached turned down the opportunity to participate in the trainings. None of the departments were compensated for these activities, other than the information and provision of barrier and safe disposal supplies; rather, all made the decision to proceed after being approached by public health actors who expressed sincere concern for the safety of the officers, as well as for the public health implications of their practices. Feedback after the training sessions suggested that both officers and SAP personnel felt that the training had been a positive and valuable experience.

In all cases the interactions necessary to design and implement the trainings also led to stronger relations between law enforcement and public health actors, including discrete innovations that link the two sectors. In Wilmington, when enrolled IDUs are arrested on charges other than paraphernalia and found to be in possession of syringes, those syringes are placed in appropriate disposal containers, which are gathered periodically by SEP personnel. Officers then issue the arrestee a voucher that he or she can take to the SEP upon release from prison or the police station to be exchanged for new sterile syringes. This sensible procedure is, to our knowledge, unique to that program and would likely not have occurred without the training and associated outreach.

Although grant funding was utilized in these three cases, it is not a necessary component of similar interventions. Trainings in Wilmington are now continuing utilizing solely police department funds, and many sources of law enforcement training funding are available that might be utilized for trainings such as those described here [56].

## Conclusion

Many harm reduction and public health actors in the U.S. remain hesitant to engage with law enforcement when planning and implementing SAPs. This reticence is understandable given the cultural and institutional differences between the two groups, but likely hampers the public health impact of these programs. Our experience has shown that police can be receptive to learning about harm reduction programs, particularly when that information is coupled with content directly relevant to the health of the law enforcement trainees and is delivered by a trusted source.

Although this feasibility work was not designed to assess the sustained impact of trainings on officer attitudes or behavior, evidence from other fields suggests that the beliefs and attitudes of officers are often heterogeneous and open to change. Properly designed training can effectively alter behavior and lead to positive effects for law enforcement and public health. The receptivity of officers to learning about harm reduction programs through this training framework supports further consideration of this model.

## Competing interests

Davis was employed within the past five years by one of the harm reduction agencies reported on in the article. Beletsky declares no competing interests.

## Authors' contributions

Both authors contributed to designing and delivering the intervention and drafting the manuscript for publication. Both authors read and approved the final manuscript.
